# Unveiling the Antibiotic Susceptibility and Antimicrobial Potential of Bacteria from Human Breast Milk of Pakistani Women: An Exploratory Study

**DOI:** 10.1155/2023/6399699

**Published:** 2023-06-19

**Authors:** Ayesha Saeed, Hina Ali, Azra Yasmin, Mehreen Baig, Abd Ullah, Abeer Kazmi, Muhammad Arslan Ahmed, Ghadeer M. Albadrani, Fatma M. El-Demerdash, Monaza Bibi, Mohamed M. Abdel-Daim, Iftikhar Ali, Sadam Hussain

**Affiliations:** ^1^Microbiology and Biotechnology Research Lab, Fatima Jinnah Women University, Rawalpindi, Pakistan; ^2^Quaid-e-Azam Medical College, Bahawalpur, Punjab, Pakistan; ^3^Surgical Unit II, Foundation University, Islamabad, Pakistan; ^4^Xinjiang Key Laboratory of Desert Plant Root Ecology and Vegetation Restoration, Xinjiang Institute of Ecology and Geography, Chinese Academy of Sciences, Urumqi, China; ^5^Cele National Station of Observation and Research for Desert-Grassland Ecosystems, Cele, China; ^6^Institute of Hydrobiology, Chinese Academy of Sciences, University of Chinese Academy of Sciences (UCAS), Wuhan, China; ^7^University of Chinese Academy of Sciences, Beijing 100049, China; ^8^Peshawar Institute of Cardiology (PIC), Peshawar, Pakistan; ^9^Department of Biology, College of Science, Princess Nourah bint Abdulrahman University, 84428, Riyadh 11671, Saudi Arabia; ^10^Department of Environmental Studies, Institute of Graduate Studies and Research, Alexandria University, Alexandria, Egypt; ^11^Department of Pharmaceutical Sciences, Pharmacy Program, Batterjee Medical College, P.O. Box 6231, Jeddah 21442, Saudi Arabia; ^12^Pharmacology Department, Faculty of Veterinary Medicine, Suez Canal University, Ismailia 41522, Egypt; ^13^Centre for Plant Sciences and Biodiversity, University of Swat, Charbagh 19120, Pakistan; ^14^Department of Genetics and Development, Columbia University Irving Medical Center, New York, NY 10032, USA; ^15^University of Health Sciences, Lahore, Punjab, Pakistan

## Abstract

**Background:**

Human life quality and expectancy have increased dramatically over the past 5 decades because of improvements in nutrition and antibiotic's usage fighting against infectious diseases. Yet, it was soon revealed that the microbes adapted to develop resistance to any of the drugs that were used. Recently, there is great concern that commensal bacteria from food and the gastrointestinal tract of humans and animals could act as a reservoir for antibiotic resistance genes. *Methodology*. This study was intended for evaluating the phenotypic antibiotic resistance/sensitivity profiles of probiotic bacteria from human breast milk and evaluating the inhibitory effect of the probiotic bacteria against both Gram-negative and Gram-positive bacteria.

**Results:**

The results point out that some of the isolated bacteria were resistant to diverse antibiotics including gentamycin, imipenem, trimethoprim sulfamethoxazole, and nalidixic acid. Susceptibility profile to certain antibiotics like vancomycin, tetracycline, ofloxacin, chloramphenicol, streptomycin, rifampicin, and bacitracin was also observed. The antimicrobial qualities of cell-free supernatants of some probiotic bacteria inhibited the growth of indicator bacteria. Also, antimicrobial properties of the probiotic bacteria from the present study attributed to the production of organic acid, bacterial adhesion to hydrocarbons (BATH), salt aggregation, coaggregation with pathogens, and bacteriocin production. Some isolated bacteria from human milk displayed higher hydrophobicity in addition to intrinsic probiotic properties like Gram-positive classification, catalase-negative activity, resistance to gastric juice (pH 2), and bile salt (0.3%) concentration.

**Conclusion:**

This study has added to the data of the antibiotic and antimicrobial activity of some probiotic bacteria from some samples of Pakistani women breast milk. Probiotic bacteria are usually considered to decrease gastrointestinal tract diseases by adhering to the gut epithelial and reducing population of pathogens and in the case of *Streptococcus lactarius* MB622 and *Streptococcus salivarius* MB620 in terms of hydrophobicity and exclusion of indicator pathogenic strains.

## 1. Introduction

Resistance to antibiotics is a worldwide health concern that is essential to be addressed from diverse perspectives. The capability of microbes to endure and flourish when exposed to antibiotics that they were initially vulnerable to is called antimicrobial resistance. Antibiotics are extensively misused and overused in humans, animals producing dairy and meat, aquaculture, and agriculture, and this unquestionably contributes to the occurrence of antimicrobial resistance [1, 2]; resistance to antibiotics can be inherent or attained [3–5]. Inheritance of intrinsic resistance is a natural antimicrobial-resistant trait presented by some probiotic bacteria. Intrinsic resistance can be explained as the nonsusceptibility of a bacterium to a known lethal concentration of antibiotic at the appropriate dose. This type of resistance typically does not compromise the safety of bacteria and is not transferable [6]. Probiotic bacteria with inherent resistance can persist very high dose of antibiotics rendering the bacteria less vulnerable to antibiotics [7]. A bacterium can attain antibiotic resistance to antimicrobial substances by getting new traits by mutations in intrinsic genes or receiving resistance genes by horizontal transfer [8]. Horizontal transfer of antimicrobial resistance genes is typically facilitated by mobile genetic components like transposons and plasmids. Antimicrobial resistance genes containing probiotic bacteria are naturally occurring that can be found in living organisms, in breast milk, and in other fermented foods [2, 6, 7, 9]. Additionally, the significant routes for the spread of antibiotic-resistant probiotic bacteria are food chain and gastrointestinal tract (GIT) [2, 10]. So these microbes can function as vectors for the transfer of antimicrobial resistance genes from foodstuff to living organisms [3, 11, 12]. Probiotics are considered as beneficial bacterial category of lactic acid bacteria, and safety of probiotics may be compromised if they function as a vector for transmittance of antimicrobial genes to potential pathogenic bacteria [6, 12]. Frequency of misusing antibiotics among women of childbearing age in developing world including Pakistan is much higher because of ineffectiveness of satisfactory guidelines on antibiotics [13, 14]. Antimicrobial resistance genes possessing probiotic bacteria could transfer those genes vertically from mother to infant during delivery or breast feeding [2, 6].

The increase in antibiotic resistance among microbes and subsequent increase in antibiotic failure to treat microbial diseases have encouraged more investigation into substitute antimicrobial compounds. Effective and favorable antimicrobial compounds are now being examined. Many studies exist on antimicrobial substances produced by lactic acid bacteria [15, 16]. The antimicrobial activity of probiotic lactic acid bacteria has been linked with production of effective metabolites including organic acids (lactic and acetic acids), hydrogen peroxide, carbon dioxide, ethanol, and bacteriocins [17, 18]. The powerful antimicrobial compounds are bactericidal against pathogenic microorganisms, thus also vital in food preservation [19, 20].

Organic acids are of great significance and effective antimicrobial compound produced by potential probiotics [19, 21]. Probiotic preparations usually comprise of probiotic bacterial strains that are acid-tolerant bacteria producing lactic acid (an organic acid) after carbohydrate fermentation as a main metabolic end-product. The antimicrobial effects caused by organic acids have been well acknowledged [22–24]. The low pH will decrease the pathogenic bacteria while assisting the propagation of the probiotic and other advantageous, organic acid-tolerant microbes in the gastrointestinal tract [25].

The main selection criterion for probiotics is their capability of adhering to the mucus produced by intestinal epithelium. This ability may raise their survival capabilities in the gut and consequently let bacteria exert their health-benefiting effects [26–29]. Though adhesion capability of probiotics does not essentially certify health benefits, their adhesion to intestinal epithelial wall can have a defensive role contrary to harmful bacteria through competition for binding sites to the host cells [30]. Commonly, adhesion is a complicated procedure comprising specific and nonspecific (hydrophobic connections between cell surfaces) ligand receptor interactions [31]. Along with that, exopolysaccharides or lipoteichoic acid manufactured by bacteria can take part in its adhesion to host epithelial cells [30, 32]. Communications between all these components are significant for adhesion in turn providing the benefits of intestine colonization of probiotic bacteria. Cell surface hydrophobicity is considered as an important functionality in general adhesion capacity. Most commonly, it is calculated by assessing the affinity of the tested strain to a hydrocarbon solvent such as the bacterial adhesion to hydrocarbon (BATH) method that measures membrane hydrophobicity of bacteria or hydrophilic nature of the cell surface. In a recent study by [33], microorganisms with elevated hydrophobicity can adhere better to epithelial cells in turn influencing the adhesion capacity. Falah et al. [34] stated that the hydrophobicity investigation can be done as a prerequisite for the adhesion capability to epithelial cells by probiotic bacteria. Hydrophobicity is considered as one of the essential properties improving the first interaction among bacteria and host cells. Aggregation (auto as well as co) and surface hydrophobicity are features that offer potential benefits for microorganisms in colonizing the gastrointestinal tract [35]. Produced by probiotic bacteria, high molecular mass antimicrobial components are strong bacteriocins and other antibacterial proteinaceous substances with narrow and broad range activity against pathogenic microbes. Among the entire techniques, agar well diffusion assay is the most used method for bacteriocin production [36].

So, we planned to evaluate the antibiotic resistance/sensitivity profile of probiotic bacteria isolated from human breast milk. The study also explores the antimicrobial activity of some of the identified probiotic bacteria to produce antimicrobial metabolites that will prevent the proliferation of pathogenic bacteria which is among the conditions for the selection of probiotic bacteria.

## 2. Materials and Methods

### 2.1. Materials

The materials used for the experimental work of this study include deMan, Rogosa, and Sharpe (MRS) agar (Merck Millipore, cat # 110660), BHI agar (BD Difco, cat # 241830), sodium hydroxide pellets (Merck Millipore, cat # 106482), ammonium sulfate (Merck Millipore, cat # 101216), and antibiotic discs (Oxoid, cat # HP0053A). Glass wear and plastic wear used in the study were purchased from Thermo Scientific.

### 2.2. Isolation and Identification

Sampling was done, by manual expression after disinfecting the skin with chlorhexidine, from healthy mothers after getting their informed consent from the local hospitals in Rawalpindi, Pakistan. Milk samples were kept on ice while transferring to the Microbiology and Biotechnology Laboratory, Fatima Jinnah Women University, Rawalpindi, for immediate isolation. The milk isolation was done on minimal media, deMan, Rogosa, and Sharpe (MRS) agar, and the distinct colonies were sent for 16S rRNA sequencing to Macrogen, Korea. Retrieved sequences were run through basic local alignment sequence tool (BLAST), and sequence of the identified strain was then submitted to the National Center for Biotechnology Information (NCBI).

### 2.3. Antibiotic Resistance/Sensitivity Profile

Antibiotic resistance of isolates was affirmed by 15 commonly used antibiotics by disc diffusion method [37]. In the management, screening, and killing of bacteria, critical role is played by antibiotics during different biotechnological processes. The vulnerability of bacteria to antibiotics is termed as antibiotic sensitivity of that specific bacterium. Clear zone which appeared around the commercially available “antibiotic disc” if bacteria are sensitive to antibiotic is called zone of inhibition. Bacteria that are resistant to antibiotic still grow around the disc.

For antibiotic sensitivity assay, MRS agar medium was prepared, autoclaved, and poured in sterile Petri plates under aseptic conditions. After solidification, plates were inverted and kept at room temperature. Three MRS agar plates were taken for each strain. Inoculum was prepared by mixing loopful of bacterial culture in 1 ml distilled water, and 50 *μ*l was spread on the surface of agar. Then, antibiotic discs of various antibiotics were placed with the help of forceps on the plates. Five discs of different antibiotics were placed on each plate, and every disc was softly pressed with the tip of forceps. The plates were incubated at 37°C. After 24 hrs, the diameter of zones of inhibition around the discs was measured in millimeters [38]. The presence or absence of bacterial growth revealed the resistance/sensitivity of bacteria against that antibiotic.

### 2.4. Antimicrobial Activity

Antimicrobial compounds (like bacteriocin, lactic acid, and hydrogen peroxide) are produced by probiotic bacteria to compete with pathogens that lead to increase the immune response of the host [39]. In vitro antagonistic assay was performed using the agar double-layer diffusion method. Bacterial isolates were spotted onto MRS agar's surface in a Petri dish and incubated at 37°C for 24 to 48 hrs. After incubation, cells were killed by exposure to chloroform for 30 min, and the residual chloroform was allowed to evaporate for another 30 min. The plates were overlaid with 3.5 ml of BHI (Difco) soft agar (0.75%) which were inoculated with 10^6^ CFU/ml of the indicator bacteria and incubated at 37°C for 24 to 48 hrs. The plates were then checked for the presence or absence of an inhibitory halo around the spot [40]. The indicator ATCC strains were obtained from Microbiology and Biotechnology Lab, Fatima Jinnah Women University, including *Bacillus subtilis* MB405, *Bacillus pumilus* MB407, *Bacillus cereus* MB401, *Alcaligenes faecalis* MB090, *Microbacterium oxydans* MB325, *Pseudomonas geniculata* MB321, *Streptomyces laurentii* MB319, *Enterococcus faecium*, *Enterococcus faecalis*, *Escherichia coli*, *Klebsiella pneumoniae* MB081, and *Staphylococcus aureus*.

### 2.5. Organic Acid Production Assay

Lactic acid bacteria are known for the production of organic acids specifically lactic acids. Isolated lactic acid bacteria were considered to have the same property, and in order to determine this ability, acid production assay was conducted [38].

Powdered skimmed milk was purchased from the local market. Autoclaved distilled water was taken and mixed with 10% of powdered skimmed milk to make sterile skimmed milk with pH 6.68. Five ml of skimmed milk was inoculated with 24 hrs fresh bacterial culture and incubated at 37°C for 24, 48, and 72 hrs. After incubation, coagulated skimmed milk was filtered, and pH of each filtrate was measured with digital electrode pH meter for lactic acid production. The filtrate was also titrated against 0.1 N NaOH, and organic acid production was quantified in terms of percentage strength [41].

### 2.6. Hydrophobicity Assay

Bacterial adherence to hydrocarbons (BATH) was performed to assess bacterial surface hydrophobicity of isolated strains. Bacterial cells from an overnight culture were harvested by centrifugation (5,000 × g, 20 min, 4°C), washed twice with phosphate-buffered saline PBS, and suspended in the same buffer. Absorbance (A600 nm) was adjusted to 0.70 ± 0.02 in order to standardize the number of bacteria (200–250 CFU/ml). The optical density (OD_600_ nm) of a homogenized bacterial suspension was recorded; then, the same suspension repeated and left to rest for 24 hrs at 37°C without vortexing. The aggregation percentage was expressed as

(1) $$\begin{array}{c} \text{BATH}\%=1-\left(\frac{A\text{Time}}{A0}\right)\times 100, \end{array}$$

where *A*Time represents the absorbance of the mixture at 24 hrs and *A*0 is the absorbance at time 0 [42].

### 2.7. Salt Aggregation Test

Salt aggregation test (SAT) was performed to assess cell surface hydrophobicity of isolated strains in complement with BATH [43]. Overnight cultures of the lactobacilli and pathogens were harvested by centrifugation at 5000 × g at room temperature. The pellet was washed twice with PBS (0.002 M, pH 6.7) and then resuspended in this buffer to a final concentration of about 1 × 10^8^ CFU/ml. Then, 25 *μ*l of the bacterial suspensions was mixed with equal volumes of ammonium sulfate at various molarities (0.2 M to 4.5 M in 0.002 M PBS with pH 6.7) on glass slide. After gentle mixing for 1 min, the lowest ammonium sulfate concentration to cause visual bacterial cell clumping was recorded as the SAT value. The SAT value is inversely proportional to the hydrophobic nature [44].

### 2.8. Coaggregation with Pathogens

Coaggregation assay was performed using the method by Collado et al. [45] with minor modifications. Overnight cultures of isolates and pathogen strains (same as used in antimicrobial assay) were washed twice with PBS (pH 6.7) and resuspended in PBS to a final concentration of 1 × 10^8^ CFU/ml. Equal volumes (1.5 ml) of isolated strains and pathogen strains were mixed, by vortexing for 10 s, and incubated at 37°C for 2 hrs without agitation. The supernatant liquids were then measured at 600 nm (A600). All experiments were performed in triplicate. Coaggregation was calculated according to the following equation:

(2) $$\begin{array}{c} \text{Coaggregation}\%=\left[1-A_{\text{mix}}\left(A_{\text{isolate}}+\frac{A_{\text{pathogen}}}{2}\right)\right]\times 100, \end{array}$$

where *A*_isolate_, *A*_pathogen_, and *A*_mix_ represent the test strains, pathogenic strains, and their mixture after incubation for 2 hrs, respectively.

### 2.9. Statistical Analysis

All the experiments were done in triplicate, and the static significance was measured using Excel statistics. The correlation among the isolates was also done using formulas from Excel software. Heatmap was constructed using R programming using ggplot and pheatmap packages.

## 3. Results

### 3.1. Isolation and Identification

From the breast milk of eleven healthy mothers, seventeen distinct colonies were sent for 16S rRNA sequence identification. Retrieved sequence was submitted to NCBI GenBank nucleotide database under the accession numbers starting from MG751364 to MG751380. Identified bacterial strains from human breast milk belong mainly from *Staphylococcus* and *Streptococcus* genera. Mainly nine identified strains were used in this study including *Staphylococcus hominis* MB606, *Staphylococcus hominis* MB613, *Staphylococcus hominis* MB614, *Staphylococcus hominis* MB615, *Bacillus* sp. MB618, *Staphylococcus hominis* MB619, *Streptococcus salivarius* MB620, *Staphylococcus epidermidis* MB621, and *Streptococcus lactarius* MB622.

### 3.2. Antibiotic Resistance/Sensitivity Profiling

The bacterial strains isolated from human milk were exposed to multiple antibiotics like gentamicin (CN 10 *μ*g), amoxicillin (AMC 10 *μ*g), erythromycin (E 15 *μ*g), streptomycin (S 10 *μ*g), imipenem (IMI 10 *μ*g), tetracycline (TE 30 *μ*g), kanamycin (K 30 *μ*g), bacitracin (BA 10 *μ*g), nalidixic acid (NA 30 *μ*g), vancomycin (VA 30 *μ*g), ofloxacin (OFX 5 *μ*g), rifampicin (RD 5 *μ*g), clindamycin (CD 2 *μ*g), chloramphenicol (C 30 *μ*g), and trimethoprim sulfamethoxazole (SXT 25 *μ*g) to study their antibiotic resistance in order to generate antibiograms for the isolates. Multiple antibiotic resistance (MAR) index was also calculated for these isolates.

**Figure 1:**
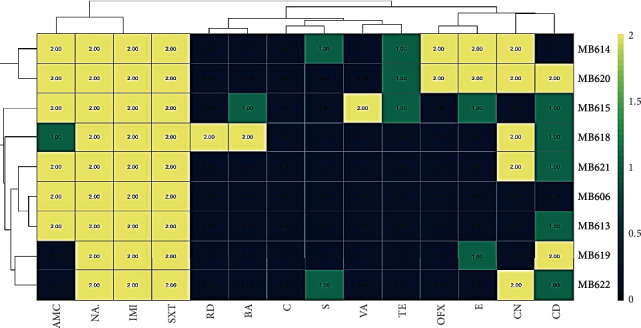
Heatmap showing the absolute abundance of antibiotic resistance or sensitivity against various antibiotics, depicting zero being most sensitive and two being most resistant.

Isolated probiotics showed sensitivity to antibiotics, and zones of inhibition of various diameters were observed against different antibiotics on Mueller-Hinton (MH) agar medium (Table 1). These zones were compared with standard zones for specific antibiotic standards mentioned in CLSC (clinical laboratory standard charts, 2007). All the probiotics from human milk were susceptible to chloramphenicol, while variation was observed for vancomycin (89%), rifampicin (89%), ofloxacin, streptomycin and bacitracin (78%), and amoxicillin (67%). All probiotics showed resistance against imipenem, trimethoprim, and nalidixic acid (Table 2). MAR index of the isolated bacteria revealed that almost all the isolates had the values ≥ 0.2 showing high resistance towards antibiotics, and the index was calculated using the formula MAR = *a*/*b* where “*a*” is the number of antibiotics to which tested isolates were resistant and “*b*” is the total number of antibiotics used in the assay (Table 3). The correlation of isolated bacteria was calculated using Excel statistics to determine the correlation coefficient among isolates against their antibiotic resistance sensitivity mechanism. The values of correlation coefficient more than 0.7 showed significance to the isolates which might be sharing the same mechanism of antibiotic resistance. The values between 0.5 and 0.7 showed moderate correlation, and values below revealed very little correlation among the stains. The correlation matrix showed that MB606 and MB613 were very closely related in terms of their antibiotic resistance/sensitivity profile and rest of the matrix showed variable results (Table 4). Heatmap summarizes the resistance and sensitivity of probiotic bacteria from human milk and grouped the strains on the basis of similarity and difference in the resistance/sensitivity profile (Figure 1).

### 3.3. Organic Acid Production Assay

**Figure 2:**
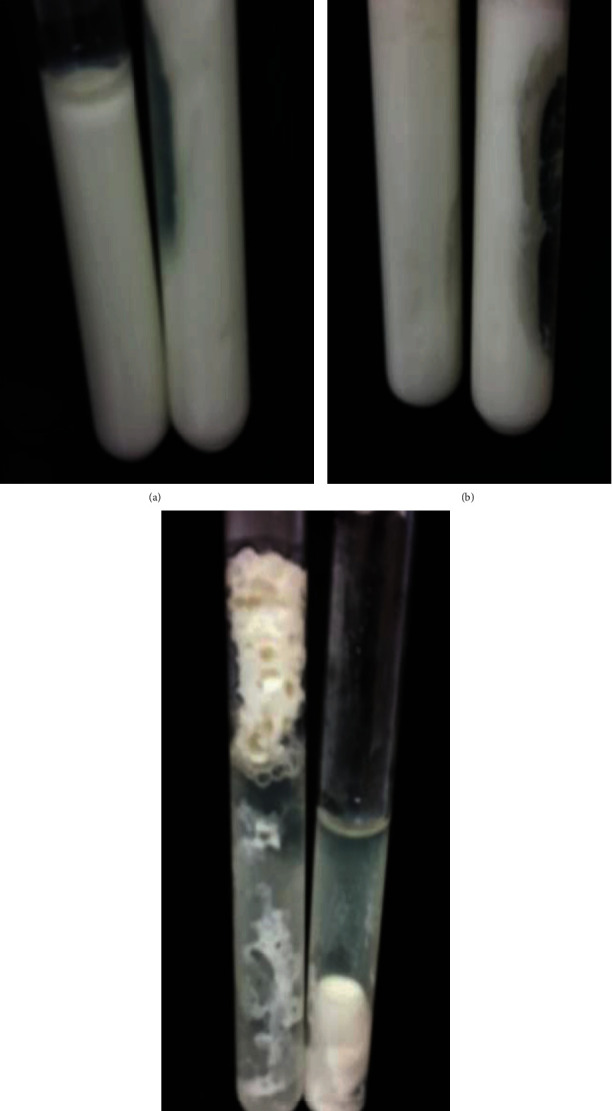
Milk coagulation by Staphylococcus epidermidis MB621 (right side) against control (left side): (a) 24 hrs, (b) 48 hrs, and (c) 72 hrs of incubation.

**Figure 3:**
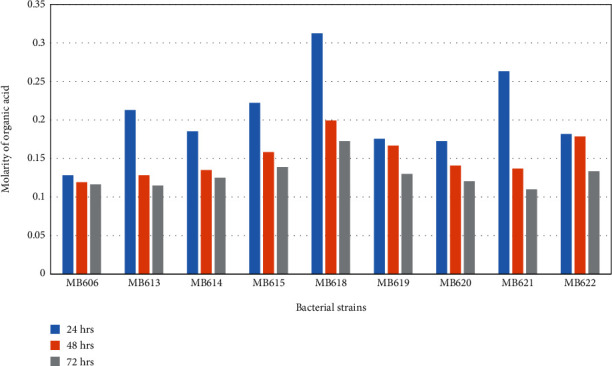
Organic acid produced by probiotic bacteria during incubation.

**Figure 4:**
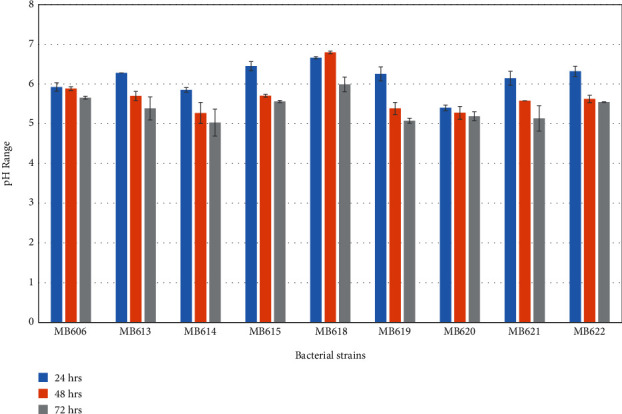
pH change during the course of incubation of skimmed milk (initial pH 7) with probiotic strains isolated from human milk.

Organic acid production ability of probiotic bacteria isolated from human milk was assayed where all the isolated probiotics were able to coagulate skimmed milk (Table 5) and produced organic acid along with a gradual decrease in medium pH (Figure 2). Bacterial strains *Staphylococcus hominis* MB606; *Staphylococcus epidermidis* MB607; *Staphylococcus epidermidis* MB608; *Staphylococcus hominis* MB609, MB610, and MB611; *Staphylococcus epidermidis* MB612; *Staphylococcus hominis* MB613, MB614, MB615, MB616, and MB617; *Bacillus* sp. MB618; *Staphylococcus hominis* MB619; *Streptococcus salivarius* MB620; *Staphylococcus epidermidis* MB621 (Figure 3); and *Streptococcus lactarius* MB622 showed decreased organic acid molarity (when titrated against 0.1 M NaOH) with the increase in time of incubation (Figure 4).

### 3.4. Hydrophobicity Assay

**Figure 5:**
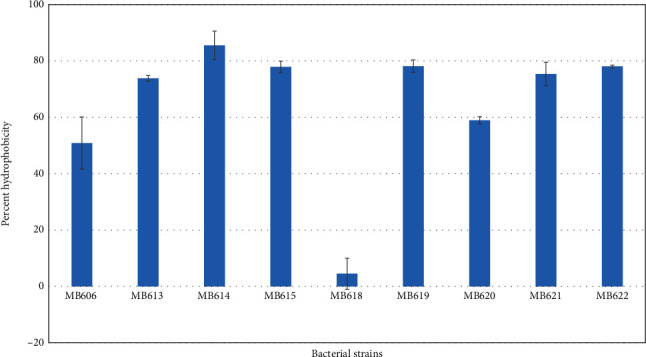
Surface hydrophobicity percentage of probiotic bacteria isolated from human milk against hydrocarbons.

To assess bacterial surface hydrophobicity, bacterial adherence to hydrocarbons (BATH) was performed. *Staphylococcus hominis* MB606, MB613, MB614, MB615, and MB619 showed 51, 74, 86, 78, and 79% hydrophobicity values, respectively. Hydrophobicity recorded for different strains was 5% (*Bacillus* sp. MB618), 59% (*Streptococcus salivarius* MB620), 75% (*Staphylococcus epidermidis* MB621), and 78% (*Streptococcus lactarius* MB622) as shown in Figure 5.

### 3.5. Antimicrobial Activity of Lactic Acid Bacteria

Antimicrobial resistance genes containing probiotic bacteria are naturally occurring that can be found in living organisms thus in their products such as in human breast milk. In order to assess the probiotic potential of some of the identified probiotic isolates from human milk, the study on antimicrobial activity of 9 selected strains was carried out. The antimicrobial activity of probiotics against 12 pathogenic bacteria was investigated, and selected strains showed variable results for inhibition of growth of all the pathogens.

### 3.6. Agar Well Diffusion Assay

Bacteriocin production activity of isolated probiotic bacteria was assayed against different available pathogenic bacteria (indicator strains) by well diffusion method on MH agar medium. Bacterial strain *Staphylococcus epidermidis* MB621 produced bacteriocin against *Bacillus subtilis* MB405, *Microbacterium oxydans* MB325, *Streptomyces laurentii* MB319, *Bacillus cereus* MB401, *E. coli*, and *Staphylococcus aureus*. *Staphylococcus hominis* MB614, *Streptococcus salivarius* MB620, and *Streptococcus lactarius* MB622 gave inhibitory zone against *Bacillus subtilis* MB405, *Streptomyces laurentii* MB319, *Bacillus cereus* MB401, and *Staphylococcus aureus* (Table 6).

### 3.7. Salt Aggregation Test

**Figure 6:**
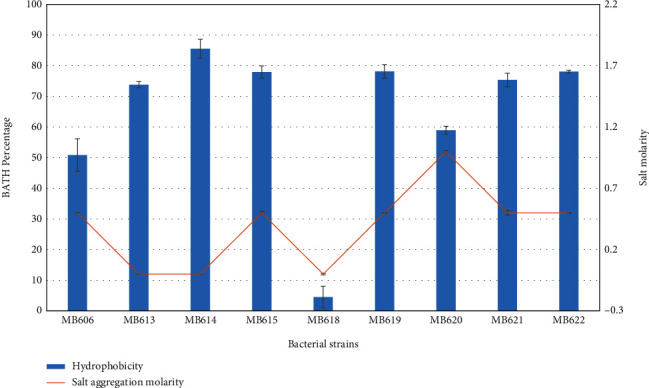
Secondary graph showing correlation among cell surface hydrophobicity and salt aggregation ability of probiotic isolates from human milk.

Salt aggregation test (SAT) proved to be a screening test for detecting bacteria with high surface hydrophobicity (Figure 6) due to surface protein of fimbrial (occurrence of fimbria protein suggestively associated with pathogenicity) and nonfimbrial nature. The isolated bacteria from human milk and the indicator strains (used previously in antimicrobial assay) aggregated in the ammonium sulfate salt solution of various molarities (0.5-4.5 M). *Streptococcus salivarius* MB620 and *Streptococcus lactarius* MB622 aggregated at 1 M and 0.5 M salt concentration, respectively (Table 7).

### 3.8. Coaggregation with Pathogens

**Figure 7:**
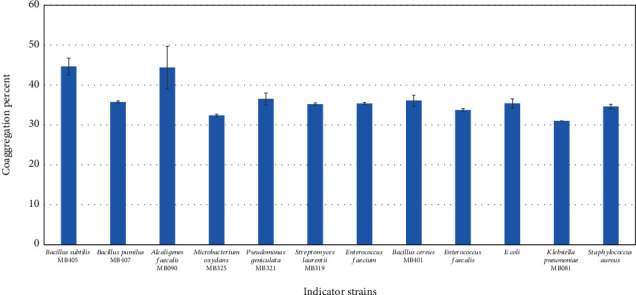
Coaggregation of Streptococcus salivarius MB620 with indicator strains.

**Figure 8:**
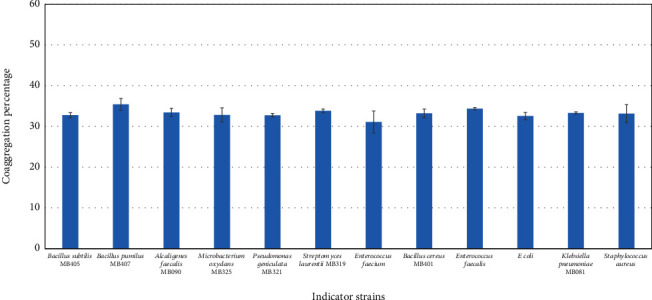
Coaggregation of Streptococcus lactarius MB622 with indicator strains.

Representative isolated probiotic bacteria (Figures 7 and 8) showed coaggregation with *B. subtilis* (33-45%), *B. cereus* (33-44%), *B. pumilus* (35-36%), and *M. oxydans* (33-37%). Among the strains tested, *Streptococcus salivarius* MB620 exhibited maximum coaggregation ability with *B. subtilis* (45%) and *B. cereus* (44%). *Streptococcus lactarius* MB622 showed the maximum coaggregation abilities with *B. pumilus* (35%). Both *Streptococcus salivarius* MB620 and *Streptococcus lactarius* MB622 showed good coaggregation activity against *E. coli*, i.e., 35% and 33%, respectively.

## 4. Discussion

In order to consider probiotic bacteria safe to be utilized in food products or as supplement, the Joint FAO/WHO Expert Committee on Food Additives Meeting and World Health Organization [46] guidelines are for the screening of antibiotic resistance/sensitivity profile. Hence, the phenotypic antibiotic resistance/sensitivity profile of probiotic bacteria isolated from human breast milk was studied. Most of the tested probiotic bacteria were sensitive to quinolones (ofloxacin) demonstrating inhibition of bacterial DNA synthesis. Also, 33% probiotic bacteria were susceptible to beta-lactams (amoxicillin), and 67% showed resistance to amoxicillin. The beta-lactams are recognized for their disruption of bacterial cell wall synthesis [47]. Furthermore, comparable to the current study, probiotic bacteria from human breast milk were susceptible to beta-lactams [6]. The probiotic bacteria were resistant to the carbapenems (imipenem), chloramphenicol, and quinolones (nalidixic acid). Similar trend was reported in the study with a total of 140 probiotics isolated from 35 kinds of Korean commercially available kimchi, where disc diffusion assay showed a resistance incidence of 98.6% for nalidixic acid [48]. Also, 78% of the isolated strains were sensitive to clindamycin, quinolones, aminoglycosides (streptomycin), and polypeptides (bacitracin), and a study with human breast milk isolates showed susceptibility to clindamycin [49]. In another study with *lactococci* isolated from dairy origin, the highest resistance frequency was observed against streptomycin, trimethoprim, nalidixic acid, and rifampicin, whereas intermediate level of resistance was seen against antibiotics like gentamycin, tetracycline, and clindamycin; susceptibility was detected against amoxicillin, erythromycin, vancomycin, ofloxacin, bacitracin, and chloramphenicol [50]. The results are mostly similar to the findings in the present study which may be because of similar origin with some difference which may be because of variation in the strains. All the probiotic bacterium isolates presented susceptibility against chloramphenicol and vancomycin (except *Streptococcus hominis* MB615) similar to the study by Sharma et al. in 2017 showing all the probiotic bacterial strains sensitive to ampicillin and vancomycin. Former reports propose inherent resistance of *lactococcal* strains to trimethoprim and cefoxitin and to the aminoglycosides—gentamicin and kanamycin [19, 51], which may somewhat clarify the resistance seen here in case of trimethoprim and other aminoglycoside drugs. Different levels of resistance to chloramphenicol, tetracycline, clindamycin, rifampicin, and erythromycin have also been reported before in *Lactococcus lactis* [19, 52]. Resistance against trimethoprim, gentamicin, erythromycin, and cephalothin for *L. lactis* strains isolated from broiler chicken feces has also been reported [53]. The variance with the current study may be because of numerous factors such as the origin of the isolates and the screening methods used. Sharma et al. [54] and Kozak et al. [49] described vancomycin resistance in *Lactobacillus plantarum* and *Lactobacillus pentosus* from human breast milk. This was also witnessed for *Leuconostoc* and *Weissella* species from fermented dairy milk [8]. In the current study, 89% of the isolates were susceptible to vancomycin. This study validates with the research finding of Jiménez et al. [55] which also stated susceptibility of *Enterococcus faecium* from human breast milk to vancomycin. The results of the current study and that of Jiménez et al. [55] suggest that the inherent resistance to vancomycin claimed for probiotic bacteria is not applicable to all species. Multidrug resistance was observed in the probiotic bacteria investigated with the highest seen in *Streptococcus salivarius* MB620, *Staphylococcus hominis* MB614, and *Bacillus* sp. MB618 which exhibited resistance to 7, 6, and 5 of the antibiotics used in this study, respectively. This trend was observed by Kıvanç et al. [56] and Reis et al. [57] who reported multiple drug resistance in *Enterococcus faecium* isolated from human breast milk to ciprofloxacin, ampicillin, gentamicin, penicillin, and vancomycin. Antimicrobial resistance is a complex problem that could be accredited to numerous kinds of mode of transmission and selection pressures [58]. Many reports have proposed that some favorable bacteria like probiotic bacteria and *Bifidobacterium* originate from the gut of a mother [59–62]. Supporting this concept, the existence of antimicrobial resistance in the probiotic bacteria tested could be linked to translocation of probiotic bacteria with these features from maternal gut to mammary glands. Maternal gastrointestinal tract and skin microflora are possible reservoirs of antimicrobial-resistant bacteria which could be transmitted to newborns and infants [49]. All of the maternal nipples were cleansed with disinfected swabs former to the collection of breast milk samples. But cleaning of breast prior to breast feeding between lactating mothers is seldom practiced in both developed and developing worlds. In probiotic bacteria, high MAR index for antibiotics as shown by the present study may perhaps be attributed to misuse of antibiotic also to the extent of antibiotic exposure to the childbearing age women and lactating mothers. Additionally, this is combined with the food chain, since once an antibiotic-resistant bacterium was ingested, it could be passed to the gut to be transferred to infants through breast milk. Native gut lactic acid bacteria have varied AMR pattern that could be transferred to other bacteria inside the gut [63]. Isolation of bacteria that are resistant to antibiotic in ready-to-eat food products specifies likelihood of spreading the bacteria from food to humans [64]. Antibiotic residues often exist in edible animal flesh and dairy products [65]. Antibiotics like tetracycline, streptomycin, penicillin, gentamicin, and erythromycin are improperly used for protection and therapeutic purpose in egg-laying poultry [66, 67]. In developing countries including Pakistan, practice of self-medication with different antibiotics is common, and antibiotics are effortlessly obtained over the counter without prescription. Lack of proper regulation and legislation add considerably to absurd antibiotic prescription and self-medication is unavoidably raising the antibiotic resistance in bacteria [68, 69].

Certain probiotic bacteria are regarded as safe by the United States Food and Drug Administration [70] due to their prehistoric use of unfermented foods and dairy. If probiotics have probability of transferring antimicrobial resistance (AMR) genes, the safety of those probiotics could be at stake [54]. Resistance to antibiotics by some probiotics in food chain has been qualified to unselective application of antibiotics [64, 71]. Antibiotic misuse by child-bearing women is also connected with global AMR [55, 72]. Furthermore, vertical transfer of AMR genes among probiotics from lactating mother to the child is likely to occur [73].

Prevailing study evaluated the resistance profile of the nine breast milk isolates against multiple antibiotics. Sensitivity of all the isolates to chloramphenicol, almost 90% to vancomycin and rifampicin and almost 80% to ofloxacin, streptomycin, and bacitracin was observed. Also, all isolates were resistant to imipenem, trimethoprim sulfamethoxazole, and nalidixic acid. However, some of the isolates were resistant to amoxicillin and gentamycin. Sensitivity of probiotic bacterial species to next-generation antibiotics from ampicillin and penicillin groups, witnessed in the present study, is consistent with other finding from human milk [54]. Resistance to vancomycin is quite common among probiotic bacteria [8, 74]. Furthermore, Kozak et al. [49] and Sharma et al. [54] described vancomycin resistance in isolated *L. plantarum* and *L pentosus* from human milk. Resistance to clindamycin, tetracycline, levofloxacin, gentamicin, and erythromycin of some probiotics as stated in the present study is consistent to the studies of other researchers [49, 54]. Constant misuse of tetracycline and erythromycin in human and in crops occurs worldwide [55, 64]. Consequently, it contributes to greater prevalence of resistance to these antibiotics and in some probiotics. According to FDA, in children with ages less than eight years, tetracycline is not suggested [75]. Therefore, the presence of some tetracycline sensitivity in almost all the isolates stated in the study is considered as less probable to impart safety risks to infants. Several investigations on human alpha-lactalbumin made lethal to tumor cells (HAMLET), a constituent of human milk harvesting favorable results on its antibiotic-potentiating influence on various bacterial species including *Staphylococci* and *Streptococci* with multiple drug resistance [76–79].

The antimicrobial activity of bacteria is important in the selection of potential probiotic bacteria. There are numerous mechanisms of action by probiotics associated with antibacterial, antifungal, antiparasitic, antiviral, anticancerous, antiallergic, and antidiabetic and enhancement of the reproductive, cardiovascular, and central nervous systems [80–82]. Antagonistic substance production by probiotic bacteria like lactic acid, bacteriocins, hydrogen peroxide, and other antimicrobial substances is linked with potential benefits of probiotic [83, 84]. Antimicrobial substances secreted by probiotic bacteria in fact have bactericidal and bacteriostatic properties on pathogenic bacteria [85, 86]. The study also surveyed antimicrobial action of probiotic bacteria against particular indicator strains. The results from agar diffusion assay using cell-free supernatant were comparatively poor in inhibition of pathogenic indicator bacteria. But *Staphylococcus hominis* MB614, *Streptococcus salivarius* MB620, *Staphylococcus epidermidis* MB621, and *Streptococcus lactarius* MB622 inhibited growth of *Bacillus subtilis* MB405, *Bacillus cereus* MB401, *Streptomyces laurentii* MB319, and *Staphylococcus aureus*. *Staphylococcus epidermidis* MB621 inhibited *Microbacterium oxydans* MB325 and *Escherichia coli.* By agar diffusion assay, the limited inhibition of pathogens was witnessed in this study which could be qualified for little concentration of antimicrobial substances along with reduced diffusion of supernatants. In [87, 88], Al-Otaibi et al. also stated reduced antimicrobial properties of probiotics from camel milk and yoghurt, in agar well diffusion assay.

Organic acid particularly lactic acid might be the utmost incompatible substance that inhibited some pathogens [89]. This study designated the proliferation of organic acid production with the time course of incubation, with a reduction in pH. Highest acid molarity of 0.3 M was observed for *Bacillus* sp. MB618; after that, 0.2 M acid production was observed in *Staphylococcus hominis* MB613 and MB615 and *Staphylococcus epidermidis* MB621; after 24 hrs of incubation at 37°C, organic acid molarity decreases during the course of incubation considerably because of other basic metabolite production during stationary phase of bacterial growth. The capability of coagulating milk when inoculated by all of the isolated strains was observed.

The antimicrobial characteristics have been accredited to the probiotic potential of isolated bacteria [90]. The study was conducted in order to evaluate the preventive properties of the isolated bacteria against both Gram-positive and Gram-negative microorganisms. Some of the probiotic bacterial strains from the study avert growth of particular indicator (pathogenic) strains by the agar well diffusion assay. The cell-free supernatants of some bacterial isolates under consideration prevented the indicator bacterial growth, showing antimicrobial abilities. Furthermore, antimicrobial properties of the isolated probiotic bacteria probably might be qualified for the organic acid (lactic acid) production, BATH, salt aggregation, coaggregation with pathogens, and bacteriocin production. This research has consequently added to the data of the antimicrobial properties of particular isolated probiotics from certain samples of Pakistani women's milk.

## 5. Conclusion

The probiotic strains were resistant to multiple antibiotics (gentamycin, imipenem, trimethoprim sulfamethoxazole, and nalidixic acid) while showing sensitivity to vancomycin, tetracycline, ofloxacin, chloramphenicol, streptomycin, rifampicin, and bacitracin. Bacterial antimicrobial behavior showed that selected probiotics were able to inhibit the growth of certain pathogens. The antimicrobial properties of probiotic bacteria may also be related to the production of lactic acid, bacterial adhesion to hydrocarbons (BATH), salt aggregation, coaggregation with pathogens, and bacteriocin production. *Streptococcus lactarius* MB622 and *Streptococcus salivarius* MB620 displayed higher hydrophobicity (78% and 59%, respectively) in addition to intrinsic probiotic properties. Probiotic bacteria are considered to safeguard gastrointestinal track (GIT) by adhering to the gut epithelial cells and reducing population of pathogens. This research has consequently added to the data of the antimicrobial activity of some isolated probiotic bacteria from some samples of Pakistani women's breast milk.

## 6. Future Applications

It would be advantageous to conduct a detailed study on the immunomodulatory properties of these bacteria from human milk. The use of starter culture with antimicrobial properties is of considerable interest due to the fact that the production of such compounds is a significant factor that helps to promote the safety and quality of fermented food. This study has financial and time constrains of investigating the potential probiotic ability to transfer antibiotic resistance gene in order to ensure their safety to be used as food supplements.

## Figures and Tables

**Table 1:** Antibiotic resistance and sensitivity profiling of various probiotics isolated from human milk against selected antibiotics.

Strains	AMC	CN	VA	TE	OFX	E	C	IMI	S	RD	SXT	CD	BA	NA
MB606	11.5 (R)	20.75 (S)	20.75 (S)	26 (S)	24.5 (S)	23.3 (S)	30.5 (S)	0 (R)	25.5 (S)	29.3 (S)	9 (R)	20.5 (S)	19.5 (S)	0 (R)
MB613	10 (R)	21.75 (S)	19.5 (S)	19.8 (S)	23.5 (S)	24.5 (S)	32.8 (S)	0 (R)	26.5 (S)	33 (S)	9 (R)	16.5 (I)	21.8 (S)	0 (R)
MB614	13.25 (R)	10.5 (R)	20.25 (S)	15.8 (I)	0 (R)	8 (R)	29.3 (S)	0 (R)	14.25 (I)	32 (S)	0 (R)	16.5 (S)	20.8 (S)	0 (R)
MB615	10 (R)	16 (S)	13.25 (R)	17.3 (I)	23.5 (S)	18.3 (I)	25.8 (S)	0 (R)	30 (S)	24.5 (S)	9 (R)	16.8 (I)	17.8 (I)	0 (R)
MB618	17.25 (I)	16.5 (R)	19.5 (S)	23.8 (S)	32 (S)	23 (S)	27.3 (S)	0 (R)	26.5 (S)	9.5 (R)	0 (R)	18.5 (I)	8.5 (S)	8.25 (R)
MB619	27.5 (S)	19.5 (S)	20.5 (S)	31.3 (S)	22 (S)	22.3 (I)	31.5 (S)	0 (R)	26.5 (S)	35.8 (S)	0 (R)	10.5 (R)	23.5 (S)	0 (R)
MB620	0 (R)	11.75 (R)	21.25 (S)	16.8 (I)	9.5 (R)	0 (R)	20.5 (S)	0 (R)	22.25 (S)	27.5 (S)	0 (R)	9 (R)	22.3 (S)	0 (R)
MB621	15.25 (R)	18.5 (R)	15.75 (S)	28 (S)	21.5 (S)	23.3 (S)	28.5 (S)	0 (R)	22.5 (S)	29.8 (S)	8 (R)	14.3 (I)	18.3 (S)	0 (R)
MB622	19 (S)	19.75 (R)	28 (S)	27.8 (S)	22 (S)	26.3 (S)	35.8 (S)	0 (R)	11.75 (I)	22.8 (S)	0 (R)	18.8 (I)	34.3 (S)	0 (R)

AMC = amoxicillin; CN = gentamicin; VA = vancomycin; TE = tetracycline; OFX = ofloxacin; E = erythromycin; C = chloramphenicol; IMP = imipenem; S = streptomycin; RD = rifampicin; SXT = trimethoprim sulfamethoxazole; CD = clindamycin; BA = bacitracin; NA = nalidixic acid; S = sensitive; R = resistant.

**Table 2:** Antibiogram for probiotic isolates from human milk.

Antibiotics	Concentration (*μ*g)	Susceptible (%)	Intermediate (%)	Resistant (%)
Amoxicillin	10	22	11	67
Gentamicin	10	44	0	56
Vancomycin	30	89	0	11
Tetracycline	30	67	33	0
Ofloxacin	5	78	0	22
Erythromycin	15	56	22	22
Chloramphenicol	30	100	0	0
Imipenem	10	0	0	100
Streptomycin	10	78	22	0
Rifampicin	5	89	0	11
Trimethoprim sulfamethoxazole	25	0	0	100
Clindamycin	2	22	56	22
Bacitracin	10	78	11	11
Nalidixic acid	30	0	0	100

**Table 3:** Multiple antibiotic resistance (MAR) index of probiotic bacteria isolated from human milk.

Strains	MAR index
*Staphylococcus hominis* MB606	0.2857143
*Staphylococcus hominis* MB613	0.321428
*Staphylococcus hominis* MB614	0.571428
*Staphylococcus hominis* MB615	0.5
*Bacillus* sp. MB618	0.5
*Staphylococcus hominis* MB619	0.321428
*Streptococcus salivarius* MB620	0.60714
*Staphylococcus epidermidis* MB621	0.392857
*Streptococcus lactarius* MB622	0.3571428

**Table 4:** Correlation matrix of probiotics from human milk in terms of their antibiotic resistance mechanism.

	MB606	MB613	MB614	MB615	MB618	MB619	MB620	MB621	MB622
MB606	**1**								
MB613	**0.959033**	**1**							
MB614	*0.592638*	0.498561	**1**						
MB615	**0.748331**	**0.755447**	0.277181	**1**					
MB618	*0.512348*	*0.517219*	0.168687	0.273861	**1**				
MB619	*0.605705*	**0.732484**	0.324065	*0.566585*	0.431016	**1**			
MB620	*0.528932*	*0.600706*	**0.795536**	0.359833	0.246361	*0.600706*	**1**		
MB621	**0.81744**	**0.843416**	*0.617431*	*0.539749*	*0.656962*	*0.588571*	*0.699422*	**1**	
MB622	*0.564288*	*0.595547*	0.494027	0.287914	*0.613267*	*0.686174*	*0.505672*	**0.789342**	**1**

Bold, positive/strong correlation; italic, moderate correlation; underline, low correlation.

**Table 5:** Milk coagulation, pH measurement, and organic acid production by probiotic bacteria isolated from human milk.

Bacterial strains	Milk coagulation	Incubation time					
		24 hrs		48 hrs		72 hrs	
		pH	Organic acid molarity	pH	Organic acid molarity	pH	Organic acid molarity
MB606	+	5.925	0.128205	5.885	0.119048	5.655	0.116279
MB613	+	6.28	0.212766	5.7	0.128205	5.385	0.114943
MB614	+	5.85	0.185185	5.27	0.134943	5.03	0.125
MB615	+	6.45	0.222222	5.705	0.158205	5.56	0.138889
MB618	+	6.66	0.3125	6.795	0.199254	5.99	0.172414
MB619	+	6.255	0.175439	5.385	0.166667	5.075	0.12987
MB620	+	5.4	0.172414	5.275	0.140845	5.19	0.120482
MB621	+	6.145	0.263158	5.58	0.136986	5.135	0.10989
MB622	+	6.32	0.181818	5.625	0.178571	5.545	0.133333

**Table 6:** Agar well diffusion assay against indicator strains.

Indicator strains	Control	MB614	MB620	MB621	MB622
*Bacillus subtilis* MB405	0	24	14	25.5	18
*Bacillus pumilus* MB407	0	0	0	0	0
*Alcaligenes faecalis* MB090	0	0	0	0	0
*Microbacterium oxydans* MB325	0	0	0	11	0
*Pseudomonas geniculata* MB321	0	0	0	0	0
*Streptomyces laurentii* MB319	0	8	10	14.5	11
*Enterococcus faecium*	0	0	0	0	0
*Bacillus cereus* MB401	0	9	9	11	8
*Enterococcus faecalis*	0	0	0	0	0
*E. coli*	0	0	0	24	0
*Klebsiella pneumoniae* MB081	0	0	0	0	0
*Staphylococcus aureus*	0	8	9	9	9

Zones were measured in millimeter around the well.

**Table 7:** SAT for probiotic isolates and indicator strains.

Probiotic isolates	Salt aggregation molarity	Indicator strains	Salt aggregation molarity
*Staphylococcus hominis* MB606	0.5	*Bacillus subtilis* MB405	0.5
*Staphylococcus hominis* MB613	0	*Bacillus pumilus* MB407	2.5
*Staphylococcus hominis* MB614	0	*Alcaligenes faecalis* MB090	0
*Staphylococcus hominis* MB615	0.5	*Microbacterium oxydans* MB325	3
*Bacillus* sp. MB618	0	*Pseudomonas geniculata* MB321	4
*Staphylococcus hominis* MB619	0.5	*Streptomyces laurentii* MB319	3
*Streptococcus salivarius* MB620	1	*Enterococcus faecium*	3
*Staphylococcus epidermidis* MB621	0.5	*Bacillus cereus* MB401	0.5
*Streptococcus lactarius* MB622	0.5	*Enterococcus faecalis*	2
		*E. coli*	1.5
		*Klebsiella pneumoniae* MB081	1
		*Staphylococcus aureus*	2.5

## Data Availability

All the data is available in the manuscript.
